# Healthcare System Digital Transformation across Four European Countries: A Multiple-Case Study

**DOI:** 10.3390/healthcare12010016

**Published:** 2023-12-20

**Authors:** Federico Fonda, Alessandro Galazzi, Stefania Chiappinotto, Linda Justi, Morten Sønderskov Frydensberg, Randi Lehmann Boesen, Mirna Macur, Erik Andrés Reig, Elisenda Reixach Espaulella, Alvisa Palese

**Affiliations:** 1 Department of Medical Science, University of Udine, Viale Ungheria 20, 33100 Udine, Italy; federico.fonda@uniud.it (F.F.); alvisa.palese@uniud.it (A.P.); 2 Health Innovation Centre of Southern Denmark, Forskerparken 10, 5230 Odense, Denmark; linda.justi@rsyd.dk (L.J.); msf@rsyd.dk (M.S.F.); rlb@rsyd.dk (R.L.B.); 3 Angela Boškin Faculty of Health Care, Spodnji Plavž 3, 4270 Jesenice, Slovenia; mmacur@fzab.si; 4 TIC Salut Social, Carrer de Roc Boronat 81, 08005 Barcelona, Catalonia, Spain; eandres@ticsalutsocial.cat (E.A.R.); ereixach@ticsalutsocial.cat (E.R.E.)

**Keywords:** multiple-case study, digital transformation, digital health, Europe, policy

## Abstract

Digitization has become involved in every aspect of life, including the healthcare sector with its healthcare professionals (HCPs), citizens (patients and their families), and services. This complex process is supported by policies: however, to date, no policy analysis on healthcare digitalization has been conducted in European countries to identify the main goals of digital transformation and its practical implementation. This research aimed to describe and compare the digital health policies across four European countries; namely, their priorities, their implementation in practice, and the digital competencies expected by HCPs. A multiple-case study was performed. Participants were the members of the Digital EducationaL programme invoLVing hEalth profEssionals (DELIVER), a project funded by the European Union under the Erasmus+ programme, involving three countries (Denmark, Italy, and Slovenia) and one autonomous region (Catalonia—Spain). Data were collected using two approaches: (a) a written interview with open-ended questions involving the members of the DELIVER project as key informants; and (b) a policy-document analysis. Interviews were analysed using the textual narrative synthesis and the word cloud policy analysis was conducted according to the Ready, Extract, Analyse and Distil approach. Results showed that all countries had established recent policies at the national level to address the development of digital health and specific governmental bodies were addressing the implementation of the digital transformation with specific ramifications at the regional and local levels. The words “health” and “care” characterized the policy documents of Denmark and Italy (309 and 56 times, 114 and 24 times, respectively), while “development” and “digital” (497 and 478 times, respectively) were common in the Slovenia document. The most used words in the Catalonia policy document were “data” and “system” (570 and 523 times, respectively). The HCP competencies expected are not clearly delineated among countries, and there is no formal plan for their development at the undergraduate, postgraduate, and continuing educational levels. Mutual understanding and exchange of good practices between countries may facilitate the digitalization processes; moreover, concrete actions in the context of HCP migration across Europe for employment purposes, as well as in the context of citizens’ migration for healthcare-seeking purposes are needed to consider the differences emerged across the countries.

## 1. Introduction

### 1.1. Digital Transformation of the Healthcare System

Digital health is defined by the World Health Organisation (WHO) as “a broad umbrella term” that includes the concept of eHealth, which is “the use of information and communications technology in support of health and health-related fields” [1]. The digital health transformation is affecting all healthcare systems and their actors across the European Union (EU). This digital transformation of health services has been described as multilevel due to it influencing care providers, services, patients and their families, and the entire healthcare system. According to its complexity, the digital transformation should be supported by appropriate policies. Starting with the advent of telematics, which embodied telecommunication and informatics to exchange data between information systems [2], digital technologies in the healthcare sector started to develop and became a fundamental factor in the delivery of healthcare. In this scenario, healthcare services can benefit from digitalization at a wide system level, where technology is seen as a tool facilitating the achievement of health-related outcomes and the fulfilment of the healthcare systems’ aims and mission [3]. In fact, digital health can be applied to a variety of patients, including children [4], adults [5], and older adults [6], as well as across a spectrum of settings including hospitals [7] and community care settings [8]. In this context, the coronavirus disease 2019 (COVID-19) outbreak has been recognized as having provided an important lesson in the value of digital solutions in healthcare settings [9]. Solutions such as video consultations, e-learning, and telemedicine provide support in managing the disease burden through planning, surveillance, testing, contact tracing, quarantine, and clinical management [10,11]. Moreover, eHealth interventions targeting healthcare professionals (HCPs) have also been implemented to improve their well-being and the quality of their work environments [12,13] to mitigate burnout and depression [14].

In the next few years, the digital transformation of health systems is expected to experience an impetus—the challenges presented by the COVID-19 pandemic [15], which forced digitalization, the limited healthcare resources in some sectors, such as nursing care, which may further trigger the digitalization process [16] as well as the innovative technological solutions available that are improving the sustainability of the healthcare sector [17], will shape and change the global models of care delivery and how we should educate and train the future healthcare workforce.

### 1.2. Digital Health Policies

Digital health policies are formal written documents that are aimed at guiding and regulating the digital transformation of healthcare and are recognized as a key bureaucratic characteristic by which modern societies function [18,19]. Digital health policies may target (a) citizens (e.g., in relation to health promotion initiatives) and patients (e.g., in relation to healthcare services), (b) healthcare providers (e.g., working with electronic medical records), (c) healthcare services (e.g., the adoption of electronic shift rostering), and (d) whole data services (e.g., all activities related to the collection, management, use, and exchange of data), including those involved in research [20].

Individual European countries are responsible for delivering health services, therefore national differences are expected. In its strategic plans, the EU, with the adoption of programs such as EU4Health 2021–2027 [21] and DIGITAL [22], has underlined the importance of health digital policies in improving the quality of care and in increasing the integration of services to deliver tailored, personalized, effective, and efficient healthcare [15]. In a resilient healthcare system, a continuous focus on digital health is required to face the new challenges of an ever-evolving healthcare system [23]. Digital health may promote the healthcare system’s sustainability; on the other hand, the sustainability of innovations introduced in their wider implications should be considered [24]. In fact, the sustainability of these digital systems is a critical aspect, as remarked by international authorities. The WHO has highlighted the need for environmentally sustainable healthcare facilities [25], capable of also providing enhanced health services with the adoption of digital health technologies, and the United Nations developed the 17 Sustainable Development Goals (SDGs) [26] as a guide towards a sustainable development, where digitalization is a target but also a factor capable of promoting sustainable development [27].

Being aware of how governments are planning digital transformations in the healthcare sector is recommended. Policy analysis can give insights into the content of policies across time, geographies, and processes [18], allowing (a) to design educational strategies targeting citizens and HCPs, (b) to redesign work processes embracing sustainability, and (c) to evaluate the emerging issues regarding how to integrate the different processes of care, namely care that can be digitalized while at the same time/simultaneously requiring personal contact [28]. In this context, to the best of our knowledge, no policy analysis of and reflection on healthcare digitalization across EU countries has been performed to date to detect the main patterns of digital transformation and implementation in practice.

Therefore, this study aims to describe and compare the digital health policies regarding (a) their priorities, (b) their implementation in practice, and (c) the digital competencies expected among HCPs to effectively implement such policies, across three European countries (Denmark, Italy, and Slovenia) and one region (Catalonia, an autonomous region of Spain); hereafter referred to as “countries”. Expanding the knowledge in this field may have multiple impacts: (a) to inform future translational policies regarding the digital health transformation by providing recommendations for undergraduate and postgraduate education; (b) to promote mutual understanding and exchange of good practices between countries that may facilitate the digitalization processes; and (c) to design concrete actions in the context of HCP migration across Europe for employment purposes, as well as in the context of citizens’ migration for healthcare-seeking purposes. The stakeholders we primarily address are the policymakers and the healthcare managers.

## 2. Materials and Methods

### 2.1. Study Design

A multiple-case study [29] was performed to provide an in-depth quality description and analysis of the digital health transformation phenomenon in a real-life context inside and across EU countries. A multiple-case study involves studying cases simultaneously or sequentially in an attempt to generate a broader appreciation of a particular issue [29]. The study was designed according to the bounded system intrinsic nature of digital transformation. The units of analysis were the policies and their implementation in practice in different countries [30] characterized by different profiles in terms of citizens, digital literacy, and usage (Supplementary Table S1), and involvement in research processes related to digital health [31]. The multiple-case study design and reporting followed the available guidelines, as summarized in Supplementary Table S2.

### 2.2. Setting and Participants

The participants were members of the Digital EducationaL invoLVing health pRofessionals (DELIVER) project, an Erasmus+ programme of the EU, which aimed to enhance the digital skills of HCPs, to support healthcare managers in the digital transformation [31], and to analyse the healthcare digitalization process as one of its intellectual outputs. The partners were the Region of Southern Denmark (Denmark), the University of Udine (Italy, the Angela Boškin Faculty of Health Care (Slovenia), and the TIC Salut Social Foundation (Catalonia, Spain). According to the Eurostat database [32], the populations of the included countries range approximately between two (Slovenia) and sixty million (Italy) citizens. The proportion of individuals with basic or above basic digital skills in 2019 ranged between 42% (Italy) and 70% (Denmark), with a European average value of 56%. Internet usage by all individuals in 2020 ranged from 78% (Italy) to 99% (Denmark) (Supplementary Table S1) [32].

### 2.3. Data Sources and Data Collection

There were two sources of data collection: (a) from within the research group, by utilizing the DELIVER project members (key informants) as individuals who participated in interviews or who completed the survey to provide information [33]; and (b) from outside of the research group, by performing a policy document data collection and analysis of the countries involved. First, a written interview based on 15 open-ended questions was developed by the members of the research project and then piloted in one country (namely, Italy) to check its feasibility and clarity. After the pilot, members were invited to participate in the first round of data collection from June to November 2021. The questions were concerned with: (a) the policies on digital health transformation of the countries involved; (b) the digital health implementation in the practice; and (c) the expected digital health competencies of HCPs. Then, data were extracted in a grid to summarize the main findings and to ensure their comparability. A second round of data collection was performed by sending all members the extracted data in May 2022. They were requested to perform a member checking of the synthesized data to validate, verify, and assess its trustworthiness [34,35]. The data were also checked monthly for accuracy during the online DELIVER project meetings.

Each member was then requested to identify the most relevant policy document addressing the digital health transformation of his/her country. This document was selected and an agreement within each country with regards to its validity and actuality was made. The documents selected were the following:

(1) For Denmark: Digital Health Strategy 2018–2022 [36] (later extended to 2024), developed by the Danish Government (88 pages, 17,375 words)
(2) For Italy: The National Recovery and Resilience Plan [37] section “Mission 6: Health”, developed by the Italian Government (13 pages, 5092 words)
(3) For Slovenia: Digital Slovenia 2020 [38] developed by the Slovenian Government (88 pages, 39,192 words)
(4) For the region of Catalonia (Spain): The Catalan Information Systems Master Plan [39], developed by the Ministry of Health of Catalonia (145 pages, 48,735 words)

The document was then sent to the Italian team, in its original format, by identifying the appropriate sections regarding the phenomenon of interest. When not available in English, the document (or its sections) was translated from the original language using an automated translating software [40] for efficiency and to provide a standardized approach. Translations were double-checked by re-sending both documents (the original one and the translation) to the members of each country who checked the validity and accuracy of the translated contents. After the translated document was approved, it was sent to the Italian team for data analysis.

### 2.4. Data Analysis

Data collected from the written interview and from the selected policy documents were exported in a table, summarised narratively, by using a textual narrative synthesis [41] method and compared in their similarities or differences.

As a secondary technique, with the intent of visualising results, we produced a cloud tag of words derived from the selected policy documents. Specifically, this word cloud analysis was based on the Ready, Extract, Analyse, and Distil (READ) approach [18], which consists of four phases applied as follows:

(a) policy documents as translated—in their selected sections were read by the research team;
(b) the words of each translated policy document regarding the digital health transformation were manually extracted and reported in a file;
(c) two researchers from each country independently examined the results and selected the words deemed to be related to digitalization and mentioned in the document at least ten times when the document had more than 100 words. In case of disagreements, a third researcher was involved. Plural-form words were collapsed into singular-form words, preserving the original meaning;
(d) subsequently, the first 100 selected words were visually displayed in a cloud tag replicating the shape of the country/region to which the document was referring through a dedicated software [42];
(e) to distil the findings, a counting technique was applied [43].

This word cloud analysis enriched the interview’s findings, as a way to highlight priorities and main topics included in each policy document, offering a visual overview allowing to detect similarities and differences across countries.

### 2.5. Rigour

The data collection process and the word cloud policy analysis were conducted by involving each member of the DELIVER team, and by sharing in multiple rounds the data analysis process and findings to ensure members were able to check them [34,35]. International rounds were performed by distance (online meetings) and face-to-face (meeting in Barcelona in 2023) whereas country-level rounds were performed by each involving at least two members. Particular attention was given to the English translation of the identified policies: the quality assessment of the translation was performed by involving members of the teams in each country to ensure rigour.

## 3. Results

### 3.1. Digital Health Transformation Policies

All study countries had an established programme or plan for digital health (Table 1) published within the last five years at the government level, following previous specific programmes in the field. In Catalonia, the specific aims of digital transformation policies have been focused on structuring mechanisms for the exchange of health information to promote the integrated functioning of the healthcare system. In addition, the programme’s scope is to enhance digital health skills among citizens and HCPs. Slovenia’s policies were intended to efficiently manage complex data and information about health, thereby reducing administrative costs. The policy has introduced effective and user-friendly digital solutions serving patients, healthcare providers, and managers. Denmark’s policies aimed to boost digital healthcare collaboration, targeting all citizens. Patients should experience the healthcare system as a coherent and trustworthy network: therefore, healthcare actors are supported in connecting patient pathways across individual interactions within the healthcare sector. Italy has been reported to be focusing on the modernization of technologies and digital screens in hospitals. The goal was to strengthen the technological infrastructure and the tools for the collection, processing, analysis, and simulation of data. Another aim was to enhance the digital skills of the HCPs. All countries had governmental bodies addressing the digital transformation, ranging from two to three regional and national bodies each (Table 1).

### 3.2. Word Policy Document Analysis

Words used in the four policy documents were analysed, and cloud tags were built (Table 2). The words “health” and “care” characterized the policy documents of Denmark and Italy (309 and 56 times, 114 and 24 times, respectively), while “development” and “digital” (497 and 478 times, respectively) were common in the Slovenia document. The most used words in the Catalonia policy document were “data” and “system” (570 and 523 times, respectively).

**Table 1:** Policies on the digital health of the countries involved.

	Denmark	Italy	Slovenia	Catalonia (Spain)
**Does the country/region have an established programme/plan for digital health?**	Yes. The Danish Digital Health strategy (national, renewed every four years). The latest document was the Digital Health Strategy 2018–2022, the first document to focus solely on digital health [36].	Yes. The first initiatives began in 2001. The latest document was the National Recovery and Resilience Plan (July 2021), which has a dedicated section regarding the digitalization of the healthcare system [37].	Yes. The Slovenian National Program eHealth was established in 2008 at the Ministry of Health. Since December 2015, e-Health activities have been transferred to the National Institute of Public Health [38]. In 2022, the new Digitalization Strategy of Health Care was adopted by the government for the period 2022–2027.	Yes. The current Catalan Health Plan 2021–2025 contains a specific area focused on digital transformation [39]. The first Health Plan was established in 1991, but digital health, as a specific area, was not included until 2016. At the national level, health matters are widely transferred to the autonomous communities, except those reserved exclusively for the Spanish Ministry of Health. There is a national strategy that is not specific to digital health: component number 19 from the National Recovery and Resilience Plan (April 2021) is specially focused on fostering digital skills.
**What was the main aim of the last programme/plan?**	To boost digital healthcare collaboration for all citizens, and the patients’ experience of the healthcare system as a coherent and trustworthy healthcare network. The strategy supports the healthcare actors in taking responsibility for interconnecting patient pathways across individual interactions with the healthcare sector.	To modernize the technology and digital screens of hospitals, to strengthen the technological infrastructure and tools for data collection, processing, analysis, and simulation. It also aims to develop and enhance the digital skills of health system personnel.	To efficiently manage complex data and information about health, as well as reduce administrative costs. Expected results of eHealth before 2027: patient-centredness, better health, trustworthy health system, integration, better accessibility.	To structure mechanisms for the exchange of health information that promote the integrated functioning of the health system; to advance digital transformation; to improve integrated care; and to enhance the health digital skills of citizens and professionals.
**Does the country/region have specific health governmental bodies that are focused on implementing digital transformation?**	Yes, five bodies: The Danish Health Authority, the Ministry of Health, The Danish Regions, Local Government Denmark, and the Agency for Digitization are the main partners in developing and implementing digital infrastructure and solutions in the Danish healthcare sector. The Agency of Digitization has a main focus on providing public ICT infrastructure, which of course is related to digital solutions in the healthcare sector (for example, MitIT).	Yes, three bodies: The General Directorate for Digitalization, the Health Information System and Statistics; more recently, the National Agency for Health Digitalization (Law 4/2022 and 25/2022).	Yes, three bodies: Digital transformation of the health system is led by the Directorate for the Digitalization in Healthcare, and the Ministry of Health.	Yes, three regional bodies: The Departmental Commission of the Coordination of Information and Communication Technologies of the Health System. The Information Systems Area of the Catalan Health System; and Information and Communication Technologies Area of the General Secretariat.

**Legend**: ICT, information and communications technology.

### 3.3. Digital Health Implementation in Practice

Different approaches are adopted by countries regarding digital implementation (Table 3). In Catalonia, the Catalan Health Service (CatSalut) annually revises service contracts with public healthcare providers, and key performance indicators are included to drive and stimulate the implementation of information and communication technology tools and services. In Slovenia, a prescriptive model is established where the government decides new digital tools and sets deadlines for their implementation. In Denmark, the implementation of national digital initiatives is mandatory. Cooperation agreements are made between the Regions, the Local Government, and the Ministry of Health, including HCPs. In Italy, the government’s new National Recovery and Resilience Plan [37] provides financial support and investments to promote digitalization. Catalonia, Denmark, and Italy leave the healthcare sector free to implement digital tools and solutions besides the ones established by the government. In each case they are obliged to be compliant with regulations, specifically the General Data Protection Regulation (GDPR) of the EU. In Slovenia, HCPs in the public sector are obliged to use the digital tools provided by the state, while the private sector might use tailored solutions. While in Denmark and Spain, there are solutions implemented throughout the national and regional levels, in Italy, solutions are available mainly at the regional level, whilst they are only at the national level in Slovenia. Supplementary Table S3 provides some examples of digital health tools implemented in hospital and in primary care settings.

### 3.4. HCP Competency Development

Countries have from 19 (Denmark) to 31 (Slovenia and Catalonia, Spain) healthcare professions defined by the law, with different bodies in charge of professional organization and competencies development (Supplementary Table S4). Slovenia, Denmark, and Italy do not currently have an established national programme/plan for improving the digital health competencies among HCPs, although the importance of digital skills was acknowledged (Table 4). In Catalonia, the COMPDIG-Salut project [44], adopted in 2020 together with the Professional Dialogue Forum, is a specific programme that aims to provide a digital skills framework and specific accreditation for HCPs. University programmes usually offer basic digital skills training in all included countries, although digital health competencies are mainly acquired on the job during clinical practice. Additionally, HCP competencies are not evaluated in a systematic manner. In Slovenia, Catalonia, and Denmark it is reported that while applying for a job position, digital competencies may be evaluated. In Italy, in the recruitment in the healthcare public sector, basic digital skills are subject to evaluation, but there are no standard evaluation procedures for digital competencies tailored to healthcare. Supplementary Table S4 provides the definitions of HCPs in the countries, along with the governmental bodies in charge of HCP-related policies, professional organization, and the maintenance and improvement of skills.

**Table 3:** Digital health implementation in practice.

	Denmark	Italy	Slovenia	Catalonia (Spain)
**How do the governmental bodies facilitate the implementation by each healthcare institution of the chosen tools, such as prescription, online consultation, etcetera?**	Being a highly digitalized country, implementation of national digital initiatives is not a choice but regarded as mandatory. Cooperation agreements are made between the Danish Regions, the Local Government, and the Ministry of Health on the one part, with the healthcare providers on the other part.	They promote initiatives such as training courses for HCPs. In addition, there is a compulsory examination at the university level which certifies basic information technology skills. Furthermore, the new Recovery Plan provides financing of telemedicine projects (teleassistance, teleconsultation, telemonitoring and tele referral) and investments to renew and reinforce hospital equipment and technology resources.	The government prescribes new digital tools and sets deadlines for their implementation.	The Catalan Health Service annually revises service contracts with public healthcare providers, and key performance indicators are included to drive and stimulate the implementation of ICT tools and services.
**Do healthcare providers have the freedom to implement additional digital tools besides those established by the government (at the national or regional level) bodies?**	Yes. Digital tools are developed and applied as needs arise in single units. For example, care homes are free to use digital tools, as long as they comply with GDPR and legal frameworks for record keeping of, for example, health information. Hospitals must comply by the local Region or on the national level through the Danish Regions. On a municipal level, more freedom is granted.	Yes. Public and private healthcare providers in specific cases can promote and implement additional innovative solutions and services in their centres as long as they are compliant with the Italian and European legal frameworks.	In some cases. Healthcare providers in the public sector (public healthcare institutions and concession holders) are obliged to use digital tools prescribed by the state. Private healthcare providers are not obliged to use them, but they represent a minority of healthcare providers.	Yes. Public and private healthcare providers have the freedom to choose and implement additional innovative solutions and services in their centres, as long as they are compliant with the Catalan, Spanish, and European legal framework.

**Legend**: GDPR, general data protection regulation; ICT, information and communications technology.

**Table 4:** Digital health competencies development for healthcare professionals.

	Denmark	Italy	Slovenia	Spain (Catalonia)
**Do institutions (higher institutions and/or universities) provide a minimum education regarding digital health?**	Yes. Universities provide some education, but it is not homogeneous. Digital health education as such is not offered.	No. At university, students are prepared in basic general digital skills not applied in the context of health. During their clinical practice, students are exposed to digital health solutions, but their use is not restricted to healthcare professionals. Therefore, their training is non-standardized in the aims and depends on the clinical instructor willing to prepare students in this topic.	No. At university, students are prepared in basic general digital skills not applied in the context of health. During their clinical practice, students are exposed to digital health solutions, but their use is not restricted to healthcare professionals. Therefore, their training is non-standardized in the aims and depends on the clinical instructor willing to prepare students in this topic.	Yes, but not homogeneously. Some universities have a course specific to digital health in the curriculum of healthcare degrees. Students are prepared in basic general digital skills.
**Does the country/region have an established programme/plan for improving the digital competencies of healthcare professionals?**	This has been acknowledged as an increasing problem and thus the need for action to enhance digital competencies has been recognized. Different initiatives address the issue. No national programme as of yet, but earmarked funding has been set up to address the need for digital skills on a national level.	There are different projects. At the national level, the Minister of Innovation and Digital Transition in collaboration with the Institute for Management Innovation in Health Care in 2020 promoted a training course named “Digital Transformation in Healthcare” for public health facilities. At the regional and local levels, programmes to train HCPs are promoted by different institutional providers and universities. The courses are often recognized as continuous medical education credits.	None focused on healthcare professionals.	The Professional Dialogue Forum organized by the Catalan Ministry of Health identified 17 professional challenges of the present and the future. Number 4 was focused on the need to improve the ICT skills of health professionals, and to advance both in the use of ICT to favour personalized care and the design of non-face-to-face care services. Following the Professional Dialogue Forum, the COMPDIG-Salut project [44] has been conceived with the aim of providing a digital skills framework and a specific accreditation for Catalan healthcare professionals. COMPDIG-Salut and the Professional Dialogue Forum are still active and working to fulfil their main objectives.
**What is your estimation regarding the digital health competencies acquired in formal courses or training, and acquired on the job?**	In most cases, competencies are acquired as peer-to-peer training. However, there are signs of a shift happening as front-runners are being trained and investments are made in continuing education in digital health.	These competencies are acquired mainly on the job. The implementation of a new digital solution (e.g., diagnostic examination booking) HCPs acquired confidence on the job.	These competencies are acquired mainly on the job. In the implementation of new digital solutions (e.g., diagnostic examination booking), HCPs acquired confidence on the job.	The digital health competencies are acquired both on the job and through training.
**Are digital competencies of HCPs evaluated? How?**	Not in any structured manner.	Digital competencies specifically tailored to the healthcare sector are not evaluated. At the university level there may be some basic ICT courses and a related exam. When joining the public sector, HCPs are enrolled via public calls, which may evaluate digital competencies at a basic level.	Digital competencies are not evaluated—perhaps by the employer at the beginning of employment. There is control over digital prescriptions for financial reasons.	To access job positions, HCPs may be asked to demonstrate general digital skills, for example by showing the Catalan Accreditation of Competencies in Information and Communication Technologies certificate, although this certification is not specific for the healthcare context [45]. The aim of the COMPDIG_Salut project is to develop an accreditation of digital competencies specific to healthcare.

**Legend:** HCPs, healthcare professionals; ICT, information and communication technology; COMPDIG-Salut, digital skills for healthcare professionals.

## 4. Discussion

To the best of our knowledge, this study is the first attempt to describe and compare the digitization transformation of the health sectors across the EU; previous analysis has been conducted in singular countries (e.g., Portugal) [46]. We performed a multiple-case study, which is a specific design capable of detecting the phenomenon in its context by involving countries at different degrees of digitalization among the citizens, from higher (e.g., Denmark) to lower (Italy), also considering the attitudes towards seeking health information using Internet sources [32]. This different baseline in digital transformation across countries may influence the attitudes of HCPs and, ultimately, the digital health transformation processes across Europe.

### 4.1. Digital Health Transformation Policies

All countries have established policy documents addressing digitization and, although, in some, the first documents were released some years ago, the process is ongoing [35,37,38,39]. From the findings, the digital transformation of the healthcare setting has been promoted in the last ten years by more policies in each country, suggesting that (a) this process is still a priority across the EU; (b) the digital transformation requires multiple and continuing efforts articulated in progressive phases; and (c) the capacity of HCPs to embrace and facilitate the expected changes is crucial.

The aims of the policy documents analysed are different across the countries, ranging from the development of pre-existent networks to data analysis and investments [35,37,38,39]. These differences are also visible in the word cloud analysis of the most frequently used words across documents, suggesting that there are different targeted priorities in place in the involved countries. Firstly, this analysis suggests that these are different in terms of quantity, with short documents (Italy) to lengthy ones (Catalonia). Second, while the priority seems to be the healthcare system in Denmark and Italy, the priorities in Slovenia and Catalonia appear to be the technological infrastructure and the process development. This seems to confirm that countries have different digital transformation achievements and needs in action; consequently, this may: (a) affect the comparison of the systems, in their healthcare functioning and outcomes; (b) require specific training strategies supporting the migration process of both HCPs and citizens; and (c) influence international cooperative projects in the field such as DELIVER [31] that should acknowledge these baseline differences while designing interventions. However, given that, except for the Italian National Recovery and Resilience Plan [37], the analysed policy documents were published just before the COVID-19 pandemic, the important advancements achieved during that time (e.g., video calls using tablets or smartphones due to hospital visiting restrictions [47,48]) may have influenced the country patterns. Therefore, continuously updating the policy analysis may inform on the priorities, and indirectly, on the digitalization implemented in the practice.

### 4.2. Digital Health Implementation in Practice

The implementation process enacted in the included countries appears to be mainly top-down: tools affecting important healthcare decisions and resources (e.g., prescriptions) are decided centrally, leaving HCPs the opportunity to propose additional tools. The healthcare sector requires standardized and interoperable solutions aimed, for example, at promoting data exchange, populating national databases, and comparing services. However, according to the findings, HCPs and institutions may provide tailored solutions [49]. Appropriate evaluation of the effectiveness and efficiency of these locally promoted tools and solutions is important to prevent redundancies and the wasting of time, and to provide a scaling-up when innovative tools may be useful on a larger scale.

### 4.3. HCP Competency Development

The importance of the digital competencies of HCPs is recognized by all countries. The role of the combined team of HCPs and information technology engineers integrating their knowledge together through a “forced marriage” has been highlighted as important for reaching the common goal of digitalization [50]. HCPs should be helped to mitigate resistance [51] by programmes aimed at developing the ability to use digital technologies in public health [10,52,53]. Nevertheless, it seems that, to date, there are no structured plans regarding the development of HCP competencies, although it is a recurrent theme in the reported aims of the analysed policy documents. In addition, there is no homogeneous training regarding digital health competencies at the undergraduate level, and the evaluation of these skills is not performed systematically or by following a common framework. A systematic review of HCP competencies has recently recommended organizational support and regular education [54]. Implementing a structured plan for the development of HCP digital competencies and a common evaluation framework is suggested. Progress has been made by Catalonia with the COMPDIG-Salut project and the Professional Dialogue Forum, which also targeted the accreditation of specific digital competencies for healthcare [36]. A higher standardized level of HCP digital competencies may benefit the wider system [51]. A national and EU strategy is advisable to ensure that HCPs can make the best possible use of information and communications technology [51], guiding them through the digitization process to deliver the best patient-tailored and evidence-based care to improve outcomes [23]. Sharing best practices, such as those implemented by Catalonia, may help in this process. However, little consideration seems to have been given to the verification of outcomes in the short and long term regarding competency development. Besides the strategic planning and implementation, there is a need to establish systems and indicators to evaluate the effectiveness of the transformations promoted.

### 4.4. Limitations

This study has several limitations. First, data collection was performed by the research team and is thus liable to information biases. However, to improve the accuracy of this multiple-case study, member checking of the survey responses was adopted [29,34,35] with several data collection rounds. Secondly, only one policy document was analysed for each country, with no historical or trend analysis. Moreover, the policy documented was selected by the team members according to its perceived importance in 2021 and this may have introduced a selection bias. Since the identification and analysis of policies, new documents may have been approved [55] or previous ones updated [56], as the digital transformation of the healthcare sector is a continuous process. Thirdly, the data collected were analysed qualitatively, according to the main intents of the study; in this context, the word cloud counting technique [43] was used to provide a visual overview of the main priorities set by the analysed policies. Therefore, the adopted qualitative methods of analysis (different techniques and quantitative-oriented data analysis) may be used in the future to quantify the findings. Fourth, the digital transformation implementation was reviewed by collecting data regarding the tools implemented in practice. The implementation process is complex and may proceed at different speeds across the country.

Therefore, the picture that emerged in this multiple-case study reflects that gained in the data collection process; due to continuous progress in the field, a continuing update of the analysis may be useful, also by the adoption of novel artificial intelligence techniques for the policy analysis.

## 5. Conclusions

The EU countries (Denmark, Italy, and Slovenia) and the autonomous region (Catalonia, Spain) participating in the DELIVER project have all established recent policies at the national level to address the digital health transition. Specific governmental bodies are addressing the implementation of digital transformation with specific ramifications at the regional and local levels. The current policies were issued in different years, suggesting that the transformation is continuous and addressed by consecutive policies based on the progressive achievements. The implementation processes seem to be mainly managed at the central level, allowing HCPs and healthcare institutions to contribute by proposing specific tools at the local level. Therefore, at the overall level, a digital metamorphosis of healthcare systems is addressed by policies with profound divergences in priorities and accomplishments pertaining to digital transformation across the involved nations. These divergent trajectories potentially obfuscate any direct intercountry comparison within the ambit of healthcare systems.

The HCP competencies expected are not clearly delineated, and there is no formal plan for their development at the undergraduate, postgraduate, and continuing education levels. Lack of education may threaten policy implementation and be a barrier to the expected outcomes in the field. Educational strategies tailored to individual national contexts are recommended given the differences across countries regarding digital transformation.

Healthcare managers should know the policies established in their own country, and also those of other countries to effectively manage newly recruited foreign HCPs. Each policy should be accompanied by specific educational strategies. We suggest a formal educational pathway defining the expected competencies and their evaluation system. Educators and professional bodies should deepen the policies to derive strategies addressing educational plans.

Researchers should support HCPs in their local attempts to provide new digital solutions to facilitate a critical evaluation of their effectiveness and to disseminate piloted experiences/best practices at the national and international levels. Further studies are recommended to compare health digitization across Europe in a wider manner by adopting a systematic approach and involving more countries.

## Figures and Tables

**Table 2:** Cloud tag of the words used in the policy documents: an analysis of frequency.

Denmark [36]		Italy [37]		Slovenia [38]		Catalonia (Spain) [39]	
** 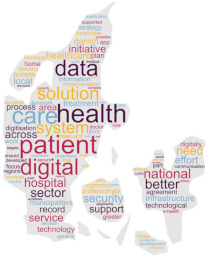 **		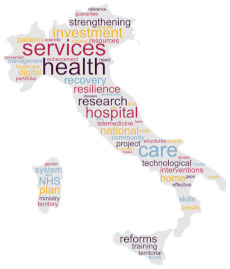		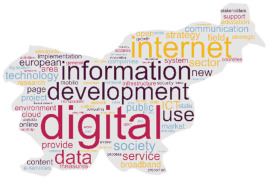		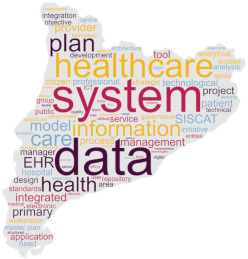	
**Words**	**n**	**Words**	**n**	**Words**	**n**	**Words**	**n**
health	309	health	56	development	497	data	570
patient	255	services	27	digital	478	system	523
system	163	national	26	Internet	307	information	482
care	114	care	24	society	270	care	471
data	109	project	19	information	268	health	455
digital	108	hospital	18	service	239	service	394
solution	87	investment	17	ICT	236	healthcare	374
treatment	81	community	16	public	212	model	333
better	67	research	15	use	208	plan	271
national	66	telemedicine	15	data	196	management	241

**Legend**: n, word frequency in the document; ICT, information and communications technology.

## Data Availability

Data are contained within the article and Supplementary Materials.

## Supplementary Materials

The following supporting information can be downloaded at: https://www.mdpi.com/article/10.3390/healthcare12010016/s1, Table S1. Demographic and digitalization data of the countries involved [32]. Table S2. Multiple-case study design checklist items [57]. Table S3. Digital health solutions in practice. Table S4. Digital health competencies development.
