# Supplementary material for: Healthcare System Digital Transformation across Four European Countries: A Multiple-Case Study
Source: Healthcare (Basel). 2023 Dec 20;12(1):16. doi: 10.3390/healthcare12010016 (PMC10778608; doi:10.3390/healthcare12010016)
Supplement: Supplementary file 1 [file healthcare-12-00016-s001.zip › healthcare-2726040-supplementary.pdf]

Healthcare

Supplementary Material

Healthcare System Digital Transformation across Four European Countries: A Multiple-Case Study

Federico Fonda [1], Alessandro Galazzi [1,*], Stefania Chiappinotto [1,*], Linda Justi [2], Morten Sønderskov Frydensberg [2], Randi Lehmann Boesen [2], Mirna Macur [3], Erik Andrés Reig [4], Elisenda Reixach Espaulella [4] and Alvisa Palese [1]

[1] University of Udine, Italy

[2] Health Innovation Centre of Southern Denmark, Denmark

[3] Angela Boškin Faculty of Health Care, Slovenia

[4] TIC Salut Social, Catalonia, Spain

[*] Correspondence: alessandro.galazzi@uniud.it; stefania.chiappinotto@uniud.it

Supplementary Table S1. Demographic and digitalization data of the countries involved.

	**Denmark**	**Italy**	**Slovenia**	**Spain**	**Europe**
**Population** on January 1st in 2021 (number)	5,840,045	59,257,566	2,108,977	47,394,223	447,007,596
**Life expectancy** at birth in 2019 (years)	79.5	81.4	78.7	81.1	78.5
**Healthy life years** expectancy at birth in 2019 (years)	58.9	68.3	60.9	69.9	64.6
People at **risk of poverty** or social exclusion in 2019 (%)	16.3	25.6	14.4	25.3	20.9
**Internet use** by all individuals in the last 3 months - 2020 (%)	99	78	87	93	88
**Internet usage** of individuals working in public administration, defence, education, human health or social work activities in 2019 (%)	99	91	96	99	97
Individuals who have basic or above basic overall **digital skills** in 2019 (%)	70	42	55	57	56
Individuals using the **internet for** seeking **health-related** information in 2020 (%)	72	46	67	58	56

Source: Eurostat 2021 [1]

Supplementary Table S2. Multiple-case study design checklist items [2].

Items	Sections of the manuscript
**1.** What is the case and its units of analysis?	Study design
**2.** Are clear objectives, preliminary research questions, hypotheses (if any) defined in advance?	Introduction
**3.** Is the theoretical basis-relation to existing literature or other cases-defined?	Introduction<br>Study design
**4.** Are the authors' intentions with the research made clear?	Introduction
**5.** Is the case adequately defined (size, domain, process, subjects…)?	Setting and participants<br>Data Collection process
**6.** Is a cause-effect relation under study? If yes, is it possible to distinguish the cause from other factors using the proposed design?	NA
**7.** Does the design involve data from multiple sources (data triangulation), using multiple methods (method triangulation)?	Rigor
**8.** Is there a rationale behind the selection of subjects, roles, artifacts, viewpoints, etc.?	Study design<br>Settings and participants
**9.** Is the specified case relevant to validly address the research questions (construct validity)?	Study design<br>Settings and participants
**10.** Is the integrity of individuals/organizations taken into account?	Rigor

Legend: NA, not applicable

Supplementary Table S3. Digital health solutions in practice.

	Denmark	Italy	Slovenia	Catalonia (Spain)
**Are there digital solutions implemented/used homogeneously at the regional/national level?**	Yes, at the national and regional level.	Yes, at the regional level.	Yes, at the national level.	Yes, at the national and regional level.
**Which are the digital tools implemented/used homogeneously throughout the country/region at the hospital level? (examples)**	The Telemedicine Ulcer Assessment Record (a national photo record to improve the quality of ulcer assessment and treatment of complex ulcers).<br>The Electronic Health Record.<br>Sundhed.dk is a national platform which serves as a singular access point to all citizen health data, including historical data and health care tools, e.g. Corona Passport, COVID-19 test results, video consultations with a therapist, list of all registered health practitioners, etc.<br>There is also a national system for medicine ordination.	The Electronic Health Record, dematerialised medical reports and medical records.	National eHealth programme offers various solutions; some of them are for patients only (EU COVID certificate, zVEM portal) and some for-health providers only (electronic registry of vaccinated people, international e-classification of diseases).<br>At hospital level they use: Central registry of Patient data; ePrescription; eAppointment and eReferral zNET (Healthcare Network Infrastructure), eHealth Users Database, Electronic Registry of Vaccinated Persons; users use zVEM portal to access their Prescriptions and referrals.	At national level: the electronic prescription of the National Health System, Patient Summary of the National Health System.<br><br>At regional level: the Clinical Health Shared Record of Catalonia, iS3, the Integrated Electronic Prescription System, Teleictus, the Catalan Personal Health Folder (“My Health”), eConsult, the Digital Medical Imaging System of Catalonia and the Anatomical pathology SIMDCAT, XatSalut.
**Which are the digital tools implemented/used homogeneously throughout the country/region at the primary care level? (examples)**	Soon to be rolled out nationally is the COPD telemedicine tool for citizens with Chronic Obstructive Pulmonary Disease, Health Insurance ID containing social security number (national), Common Medicine card (national, COVID-19 Passport (national app), in addition to private	There are no tools used and implemented homogeneously throughout the country/region at the primary care, except of the Electronic Health Record and the dematerialised medical reports. HCPs, in particular the general practitioners, can adopt their own electronic tools for charting.	On the national level all the above solutions are implemented also in primary health care level as well as Family medicine model practices, which supports protocols for the treatment of chronic patients, extended and regular preventive screenings, the establishment of registers	At national level (not an exhaustive list): the electronic prescription of the National Health System, Patient Summary of the National Health System.<br><br>At regional level (not an exhaustive list):<br>The Clinical Health Shared Record of Catalonia, iS3, the

	apps provided by general practitioners used for appointments, video consultations, etc.		of chronic patients, and the assessment of treatment quality by means of quality indicators.	Integrated Electronic Prescription System, the Catalan Personal Health Folder (“My Health”), eConsult, the Digital Medical Imaging System of Catalonia, XatSalut, eCAP, App STOP COVID-19 CAT, App GestioEmocional.cat, Salut Respon.

Legend: EU, European Union; COVID-19, Coronavirus disease 2019; COPD, chronic obstructive pulmonary disease, ID, identification, HCPs, healthcare professionals

Supplementary Table S4. Digital health competencies development.

	Denmark	Italy	Slovenia	Catalonia (Spain)
**Do the country/region has a specific definition of healthcare professionals?**	Yes. The Danish Health and Medicines Authority's concept database states that a health professional is a health actor who is affiliated with a health-producing unit. There are 19 registered health care professions.	Yes. In general, a health professional is a practitioner who provides preventive, curative, diagnostic or rehabilitative health care services systematically to individuals, families or communities. There are 22 healthcare professions.	Yes. Healthcare professionals are defined in a Law of healthcare activity. There are 31 health care professions.	Yes. Defined by the Spanish Law there are Healthcare Professions (*Profesiones sanitarias tituladas*) which are differentiated from Healthcare Vocational Training Professionals (*Profesionales área sanitaria de formación profesional*). There are 16 healthcare professions and 15 healthcare vocational training professionals.
**Examples of Health Care Professionals?**	Nurses, Midwifes, Physicians	Nurses, Physicians, Physiotherapists	Nurses, Physicians, Physiotherapists	Nurses, Physicians, Physiotherapists
**Which department /governmental bodies are in charge of healthcare professionals (with functions related to policies in the field of a professional organization, professional development and the maintenance/ improvement of skills)?**	The Ministry of Higher Education and Science is responsible for this area. Also, the Danish Patient Safety Authority (legal minimum standard for healthcare practitioners), RKKP (an example of the Regions clinical quality development programme), and the Institute for Quality and Accredition in the healthcare system (IKAS). All on national level.	There are two main institutions:<br>1. Directorate-General of Health Professions and Human Resources of the National Health Service.<br>2. Healthcare Professionals Boards (e.g., College of Nurses, College of Physicians)	The health care sector is managed by the Ministry of Health. Each profession in health care has its own chamber responsible for professional development, licenses and improvement of professional skills. The Nurses and midwife Association of Slovenia is the national regulatory authority for the field of nursing and midwifery. The Medical Chamber of Slovenia is national regulatory authority for medical doctors.	At regional level:<br>1. Directorate general of Healthcare professionals of the Catalan Health Department.<br>2. The Catalan Colleges of Healthcare Professions (e.g.,Catalan Council of Colleges of Nurses, Catalan Council of Colleges of Physicians, College of Physiotherapist)
**At which level?**	KiAP (regional) is the organization for the general practitioners usually working with quality development within a cluster-based system.	The Directorate-General of Health Professionals and Human Resources of the National Health Service is based in Rome and has national competence. Healthcare Professionals Board are instituted	On national level.	At national level:<br>1. Directorate General of Professional Regulation (*Dirección General de Ordenación Profesional*).<br>2. The Healthcare Professions Councils such as: General

Recently, the term “health clusters” was introduced, covering groups of healthcare actors from various sectors (hospitals, general practitioners, rehab centers, elderly care/nursing homes, etc.). This is a collaborative agreement between The Government, Danish Regions and Local Government Denmark.	on a national, regional and provincial level.		Council of Nurses, General Medical Organization, General Council of Physiotherapist, etc.

References

1. Eurostat - European Commission. Eurostat. Available online: https://ec.europa.eu/eurostat. (access on 15 December 2023).
2. Runeson, P.; Höst, M. Guidelines for conducting and reporting case study research in software engineering. *Empir Software Eng*. **2009**, 14, 131–164.
